# Book Notices

**Published:** 1882-09-20

**Authors:** 


### *BOOK NOTICES.*

PRACTICAL MEDICAL ANATOMY.—A guide to the Physician in the study of relations of the viscera to each other in health and disease, and in the diagnosis of the Medical and Surgical conditions of the Anatomical structures of the head and trunk, By Ambrose L. Ranney, A.M., M.D.; Adjunct Professor of Anatomy, and late Lecturer of genito urinary, and Minor Surgery in the Medical Department of the University of the city of New York—lale Surgeon to the Northern and Northwestern Dispensaries ; resident fellow of the New York Academy of Medicine ; member of the Medical Society of the county of New York ; author of the applied Anatomy of the nervous system etc. etc. New York : Wm. Wood & Co., 1882. McGarrity & Laird Agents, Atlanta, Ga.

The above is an illustrated work of 339 oc. pages neatly gotten up. Its title page, above quoted, sufficiently indicates the scope and practical importance of the work to the practitioner. "It has been written, says the author, for use of the general practitioner in his daily practice, with the hope that it might present to him the study of Anatomy from the stand point of its general interest and practical utility, and afford him the means of refreshing certain points which can be constantly applied without entailing upon him descriptive detail."

THE CHANGE OF LIFE IN HEALTH AND DISEASE.—A Chemical Treatise on the Diseases of the Ganglionic Nervous system incidental to women at the Decline of Life. By Edward John Tilt, M.D. Past President of the Obstetric Society of London. Fourth edition. Philadelphia : P. Blackston, Son & Co., 1012 Walnut street : 1882.

The subjects of the above work are very meagerly and imperfectly treated in our usual authorities, and are indeed very little understood by the Profession generally ; and yet they are of great importance, and ought certainly to be studied by every practitioner. We can commend the above work as it contains a fund of information which the Physicians in daily practice can ill afford to be without.

ESSENTIALS OF VACCINATION.—A compilation of facts relating to vaccine innoculation and its influence in the prevention of small-pox, by W. A. Hardaway, M. D., Professor of Diseases of the Skin in the post graduate faculty of the Missouri Medical College, St. Louis; Member of the American Dermatological Association; formerly one of the vaccine physicians to the city of St. Louis. Chicago: Jansen, McClurg & Co., 1882. A work of 146 pages, cloth, large type, plain and neatly published.

This work is timely and necessary, and will meet a want long felt by the general practitioners throughout the country who have but little acquaintance with the history and results of vaccination. Here they are furnished with a snug little volume giving a careful compilation of all the essential facts relating to the subject. We commend it to the profession.

